# Parents’ Self-Reported Versus Child Evaluation of Parents’ Mental Health Outcomes: Is There a Difference

**DOI:** 10.1093/geroni/igab046.767

**Published:** 2021-12-17

**Authors:** XinQi Dong, Yingxiao Hua, Dexia Kong, Qun Le

**Affiliations:** 1 Rutgers University, Rutgers Institute for Health, New Jersey, United States; 2 Rutgers University, New Brunswick, New Jersey, United States; 3 The Chinese University of Hong Kong, Hong Kong , Hong Kong

## Abstract

Older Chinese-Americans are more likely to experience depressive symptoms compared to the general U.S. aging population. This paper aims to examine the level of congruence between parents’ self-reported mental health and children’s evaluation of their parents’ mental health. Dyad-level understanding is particularly relevant considering the family-based medical decision-making preference in the Chinese-community. Older parents’ depressive symptoms were measured by PHQ-9 with a cutoff of 5 indicating the presence of depressive symptoms. Adult children were asked whether their parents informed them of their depressive symptoms or if they suspected that their parents were depressed. Logistic regressions were conducted. Parents’ self-reported depressive symptoms were associated with both adult children’s awareness (OR:3.28 (2.00-5.39)) and suspicion (OR:3.10 (2.02-4.77)) of their parents’ depressive symptoms. Results remained consistent among mother-child and father-child dyads. Study findings underscore the importance of incorporating adult children’s’ perspective in mental health research in the Chinese community.

